# Immobilization of *Brassica oleracea* Chlorophyllase 1 (BoCLH1) and *Candida rugosa* Lipase (CRL) in Magnetic Alginate Beads: An Enzymatic Evaluation in the Corresponding Proteins

**DOI:** 10.3390/molecules190811800

**Published:** 2014-08-07

**Authors:** Chih-Hui Yang, Chih-Chung Yen, Jen-Jyun Jheng, Chih-Yu Wang, Sheau-Shyang Chen, Pei-Yu Huang, Keng-Shiang Huang, Jei-Fu Shaw

**Affiliations:** 1 Department of Biological Science & Technology, I-Shou University, Kaohsiung 840, Taiwan; 2 Department of Biomedical Engineering, I-Shou University, Kaohsiung 840, Taiwan; 3 The School of Chinese Medicine for Post-Baccalaureate, I-Shou University, No.8, Yida Road, Jiaosu Village Yanchao District, Kaohsiung 82445, Taiwan

**Keywords:** *Brassica oleracea* chlorophyllase 1, *Candida rugosa* lipase, immobilization, alginate, magnetic beads

## Abstract

Enzymes have a wide variety of applications in diverse biotechnological fields, and the immobilization of enzymes plays a key role in academic research or industrialization due to the stabilization and recyclability it confers. In this study, we immobilized the *Brassica oleracea* chlorophyllase 1 (BoCLH1) or *Candida rugosa* lipase (CRL) in magnetic iron oxide nanoparticles-loaded alginate composite beads. The catalytic activity and specific activity of the BoCLH1 and CRL entrapped in magnetic alginate composite beads were evaluated. Results show that the activity of immobilized BoCLH1 in magnetic alginate composite beads (3.36 ± 0.469 U/g gel) was higher than that of immobilized BoCLH1 in alginate beads (2.96 ± 0.264 U/g gel). In addition, the specific activity of BoCLH1 beads (10.90 ± 1.521 U/mg protein) was higher than that immobilized BoCLH1 in alginate beads (8.52 ± 0.758 U/mg protein). In contrast, the immobilized CRL in magnetic alginate composite beads exhibited a lower enzyme activity (11.81 ± 0.618) than CRL immobilized in alginate beads (94.83 ± 7.929), and the specific activity of immobilized CRL entrapped in magnetic alginate composite beads (1.99 ± 0.104) was lower than immobilized lipase in alginate beads (15.01 ± 1.255). A study of the degradation of magnetic alginate composite beads immersed in acidic solution (pH 3) shows that the magnetic alginate composite beads remain intact in acidic solution for at least 6 h, indicating the maintenance of the enzyme catalytic effect in low-pH environment. Finally, the enzyme immobilized magnetic alginate composite beads could be collected by an external magnet and reused for at least six cycles.

## 1. Introduction

Despite the wide variety of applications of enzymes in diverse biotechnological fields, the utilization of their free forms are usually not cost-effective, because in general the enzymes were not recyclable after the catalytic reaction, and are not suitable to be used in industrial or mass catalysis processes [1,2,3]. Enzyme immobilization, a key topic for industrialization by stabilizing enzymes and making them recyclable, is one of the feasible approaches that can overcome the abovementioned limitations [4,5]. However, due to the possible loss of enzyme activity during the immobilization, preserving the activity of the immobilized enzyme becomes quite an important issue [1]. In the literature, several approaches have been used to immobilize enzymes on solid carriers, such as physical adsorption [6,7], encapsulation [8], covalent binding [9,10], spacer arms [11,12], *etc.* Compared with other immobilization methods, enzyme encapsulation (*i.e.*, the enzyme is entrapped in the internal structure of a polymer) possesses several advantages such as higher preservation of catalytic activity, better mobility that can enhance the enantioselectivity, and improved thermal stability and operational stability that result in higher catalytic conversions [4]. However, entrapping the enzymes in gels has some drawbacks, including potential diffusion limitations related to the substrate reaching the enzyme.

Entrapment of enzymes in alginate polymer is one of the conventional methods of enzyme immobilization. It is a technique to retain the enzyme while allowing penetration of substrate. Alginate is a nontoxic, inexpensive and versatile material and it shows thermostable properties and improved enzyme stability and functional properties [13,14,15,16,17]. Furthermore, alginate itself is well preserved in a low pH environment, allowing the catalytic process of the immobilized enzyme to be conducted in acidic solution. On the other hand, the superparamagnetic iron oxide (Fe_3_O_4_) nanoparticles are an emerging technology and they can be used in enzyme immobilization for recycling and reuse [18]. The superparamagnetic iron oxide nanoparticles–loaded polymer beads can be collected by an external magnetic field very rapidly and efficiently. Therefore, the magnetic iron oxide nanoparticles (MIO NPs) were widely used in collection, concentration and/or recycling [19,20].

**Scheme 1:**
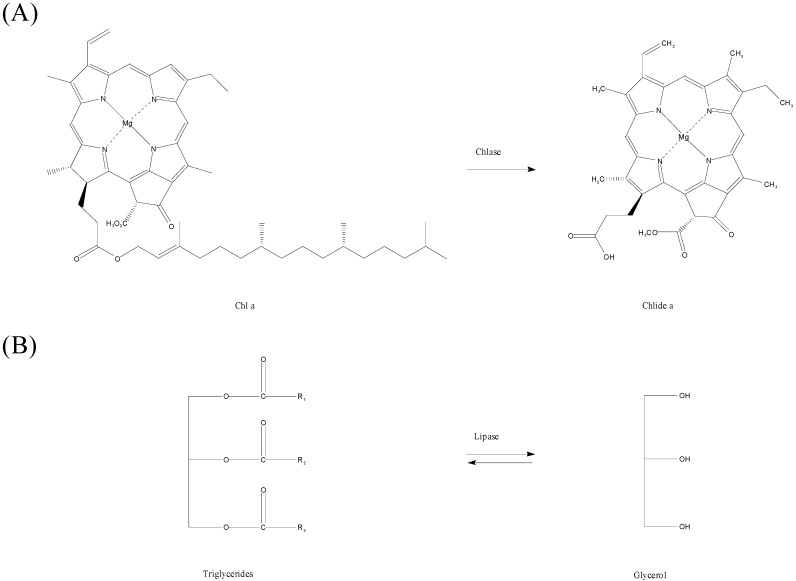
Enzymatic reaction of Chlorophyllase (CLH) andlipase. (**A**) Chlorophyllase catalyzes the hydrolysis of chlorophyll a (Chl a), chlorophyllide a (Chlide a), and phytol. (**B**) Lipase catalyzes hydrolysis or synthesis of a triglycerol.

Chlorophyllase (chlorophyll chlorophyllidohydrolase, Chlase, EC 3.1.1.14), is the first enzyme of chlorophyll (Chl) degradation, which catalyzes hydrolysis of Chl to chlorophyllide (Chlide) and phytol (Scheme 1A) during the degreening processes of plants such as leaf senescence, pathogen infection, and fruit ripening [21,22]. In industry, Chlase has been used to remove the high content of Chl from canola oil to avoid a decrease of oxidative stability [23]. Moreover, previous research indicated that treated Chlide and phytol in cell culture medium can retain their antioxidant, anticancer, antivirus, anti-inflammatory and antibacterial functions *in vitro* [24,25,26,27,28,29]. Therefore, Chlase is a potential enzyme for industrial applications.

*Candida rugosa* lipase (CRL) is a famous extracellular lipase that is a very important industrial enzyme widely used in biotechnological applications [30,31,32,33,34,35]. The CRL catalyzes the hydrolysis or synthesis of lipids (Scheme 1B). The reaction of CRL is an equilibrium reaction, and the ester synthesis occurs under non-aqueous conditions [36]. Potential industrial applications of CRL include production of free fatty acids and glycerol via hydrolysis of lipids, modification of triglyceride mixtures’ composition and physical properties by esterification, and synthesis of artificial chemicals in organic solvents [35]. In previous work *Brassica oleracea* Chlase 1 (BoCLH1) was successfully isolated and expressed in *Escherichia coli* [37]. Commercial CRL has become available for industrial uses and has been exploited in several research studies for deeper understanding of the enzyme [38].

**Figure 1:**
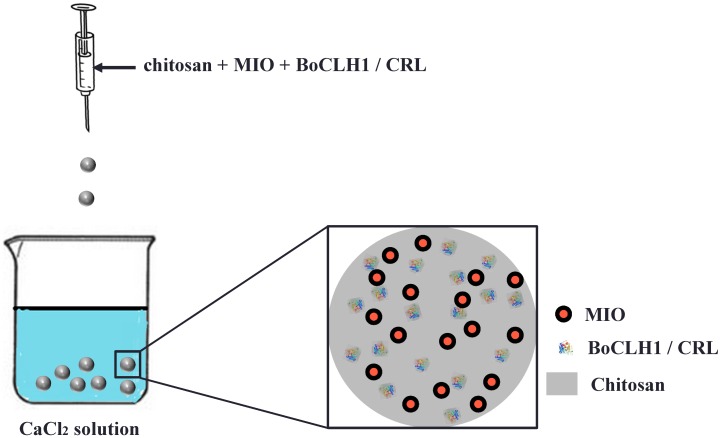
Schematic diagram showing the synthesis of enzyme encapsulated magnetic alginate composite beads. The MIO is magnetic iron oxide (Fe_3_O_4_ nanoparticles); The BoCLH1 is *Brassica oleracea* chlorophyllase 1; The CRL is *Candida rugosa* lipase.

Alginate was well known support used to entrap enzymes. Enzymes entrapped in alginate show improved stability and functional performance [13,14,15,16,17]. MIO NPs are an emerging science and they can be used in enzyme immobilization for recycling and reuse. Previous studies indicated the enzyme cross-linked onto Fe_3_O_4_ MIO NPs exhibited better stability [39,40], therefore, the purpose of this study was to investigate the immobilization yield and biocatalysis of BoCLH1- or CRL-loaded magnetic composite beads. The synthetic process for preparation of the MIO NP-loaded alginate composite beads is shown in Figure 1.

## 2. Results and Discussion

### 2.1. Morphology

**Figure 2:**
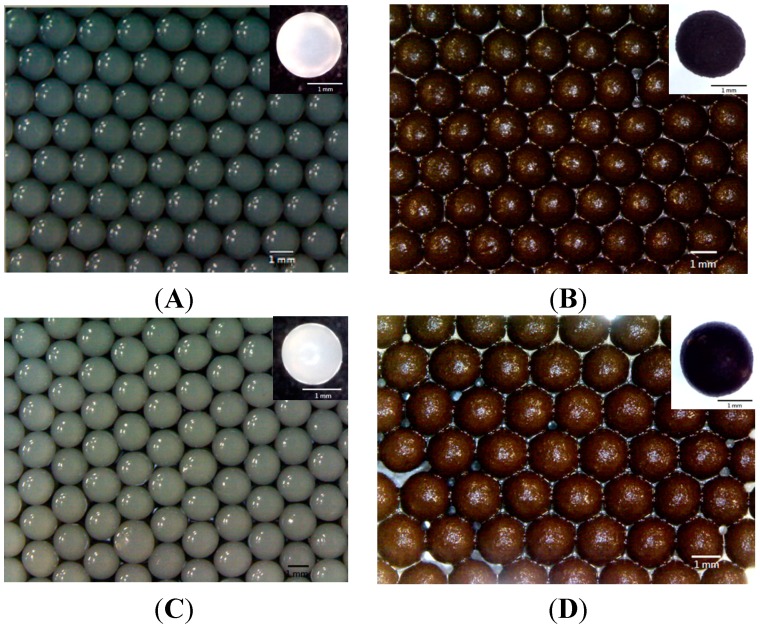
The photo images of alginate beads and alginate composite beads. (**A**) Alginate beads. (**B**) The MIO NPs loaded alginate beads. (**C**) Immobilized BoCLH1 alginate beads. (**D**) Immobilized BoCLH1 magnetic alginate composite beads.

Figure 2A shows the pure gray colored alginate beads with an average diameter of 1.8 mm. Figure 2B shows the MIO NP-loaded alginate beads with an average diameter of 2.5 mm. The MIO NP-alginate composite beads are black due to the Fe_3_O_4_ NPs inside. Figure 2C shows that the immobilized BoCLH1 alginate beads are with a lighter gray color at the surface, and their average diameter is 1.6 mm. Figure 2D shows the immobilized BoCLH1 magnetic alginate composite beads. Both the MIO NPs and BoCLH1 are loaded in the same beads. The color of the beads is black, indicating the existence of Fe_3_O_4_ NPs.

**Figure 3:**
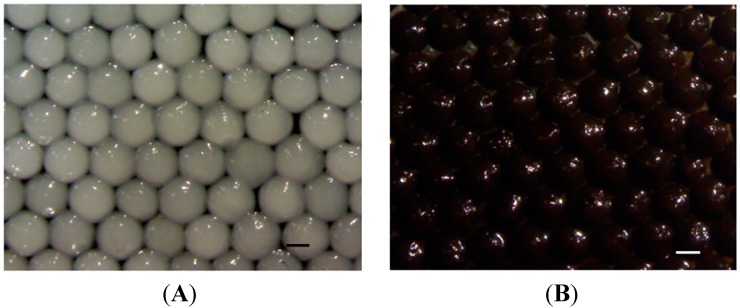
The photo images of immobilized CRL alginate composite beads (**A**) without MIO NPs and (**B**) with MIO NPs.

Figure 3 shows the immobilized CRL alginate beads without (Figure 3A) and with (Figure 3B) MIO NPs. The immobilized CRL alginate beads show a gray color and an average diameter of 2 mm. In addition, the size of the immobilized CRL magnetic alginate composite beads is 1.8 mm in diameter with black color. The beads shown in Figure 2 and Figure 3 are spherical and with uniform colour. This result presumes the MIO NPs and the BoCLH1/CRL are monodispersed inside each bead. The relative standard division of beads shown in Figure 2 and Figure 3 is below 10%, meaning the prepared beads are uniform in size.

### 2.2. pH Sensitive Reactions of the Prepared Alginate Composite Beads

**Figure 4:**
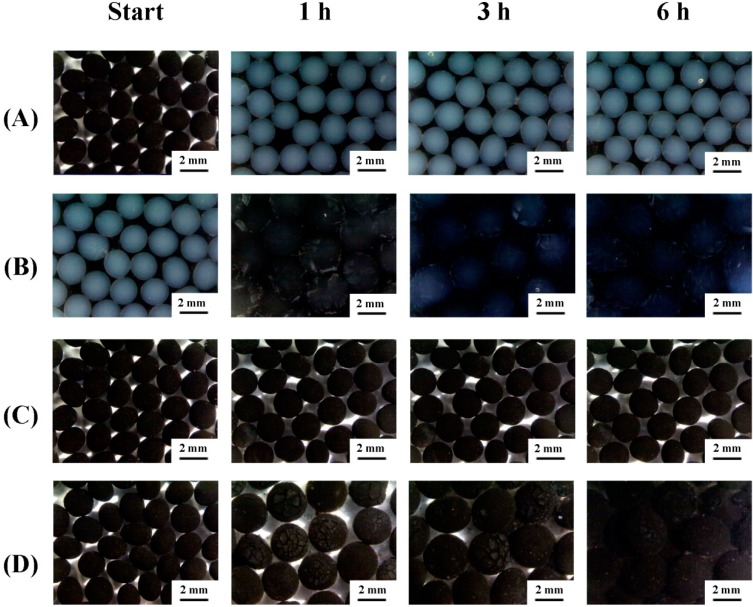
The degradation of alginate beads in various pH environments.

Figure 4A shows the alginate beads immersed in acidic solution (pH 3). The results show that alginate beads did not degrade in such acidic solution for at least 6 h. Figure 4B shows the degradation of alginate beads immersed in neutral solution (pH 7). The alginate beads started to disintegrate during the first hour, and degraded gradually with time. Figure 4C shows the MIO NP-entrapped alginate beads immersed in acidic solution (pH 3). The results show that the MIO NP-entrapped alginate beads remained intact in acidic solution for at least 6 h. Figure 4D shows the degradations of the MIO NP-entrapped alginate beads immersed in neutral solution (pH 7). The MIO NPs-entrapped alginate beads swelled gradually with time. In the sixth hour, alginate beads become more swollen, and started to disintegrate. We speculate that when MIO NPs are employed, the structure of beads become rigid. These results show that the alginate beads and alginate composite beads have excellent acid-resistance characteristics. Thus, enzymes immobilized in alginate beads are able to stay intact in the acidic environment and still can maintain their catalytic effect characteristics. In addition, the MIO NP- entrapped alginate beads have further advantages, such as staying intact in a neutral environment for longer time and being recyclable and reusable.

### 2.3. The Enzyme Entrapped and Immobilization Yield

The enzyme entrapped and immobilization yield of BoCLH1 and CRL alginate composite beads are summarized in Table 1. To investigate the effects of the MIO NPs content on the entrapped enzyme, inclusion of BoCLH1 and CRL in alginate was 10.45 mg and 6.32 mg, which more than in MIO NPs-alginate beads which was 9.26 mg and 5.93 mg, respectively.

Immobilization yield (%) was defined as follows:

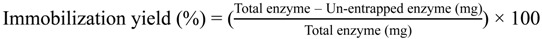
(1)


The immobilization yield of BoCLH1 and CRL of alginate beads (70% and 63.2%) was more than that of MIO NPs-alginate beads (62% and 59.3%). These results suggest that the presence of the MIO NPs decreases the amount of entrapped enzyme.

**Table 1:** Immobilization efficiency of BoCLH1 and CRL entrapped in alginate beads or MIO NPs-alginate beads.

Samples	Enzyme Entrapped (mg/g gel)	Immobilization Yield (%)
Free BoCLH1	0	ND
BoCLH1 in alginate	10.45	70%
BoCLH1 in MIO NPs-alginate	9.26	62%
Free CRL	0	ND
CRL in alginate	6.32	63.2%
CEL in MIO NPs-alginate	5.93	59.3%

ND: Not detected.

### 2.4. The Immobilized Enzymes Catalytic Activity and Specific Activity

The catalytic activity and specific activity of the BoCLH1 and CRL entrapped in magnetic alginate composite beads are shown in Table 2. The free and immobilized BoCLH1 activity assay was performed according to the procedure recommended in Lee *et al.* [34]. The enzyme activity of immobilized BoCLH1 in alginate was 2.96 ± 0.264 U/g gel. The enzyme activity of BoCLH1 entrapped in MIO NPs-alginate composite beads was 3.36 ± 0.469 U/g gel. The specific activity of BoCLH1 entrapped in alginate or in MIO NP-alginate composite beads was about 2.31–2.96-fold higher than that of free BoCLH1. This result could be attributed to the fact this immobilization preserves the mobility of the BoCLH1 and allows its activity to increase.

**Table 2:** Hydrolytic activity of entrapped BoCLH1 and CRL in alginate beads or MIO NP-alginate composite beads.

Samples	Enzymatic Activity (U/g gel)	Specific Activity (U/mg protein)
Free BoCLH1	ND	3.68 ± 0.027 (100%) ^a^
BoCLH1 in alginate	2.96 ± 0.264	8.52 ± 0.758 (231.5%)
BoCLH1 in MIO NPs-alginate	3.36 ± 0.469	10.9 ± 1.521 (296.2%)
Free CRL	ND	60.78 ± 1.311 (100%)
CRL in alginate	94.83 ± 7.929	15.01 ± 1.255 (24.7%)
CRL in MIO NPs-alginate	11.81 ± 0.618	1.99 ± 0.104 (3.3%)

ND: Not detected; ^a^ Data in parentheses represent the relative activities (%) of each enzyme entrapped in in alginate beads or MIO NPs-alginate. The activity of free enzyme is denoted as 100%.

The esterase activities of free and immobilized CRL were measured by a previously described method using *p*-nitrophenol butyrate as the substrate [35]. However, contrary to the entrapped BoCLH1, the CRL entrapped in alginate exhibited a higher enzyme activity (94.83 ± 7.929 U/g gel) than in alginate containing MIO NPs (11.81 ± 0.618 U/g gel). Compared with free CRL, the specific activity of CRL entrapped in alginate or in MIO NP-alginate composite beads remained at 24.7% and 3.3% activity, respectively.

### 2.5. pH Effects of Immobilized BoCLH1 and CRL Activity

**Figure 5:**
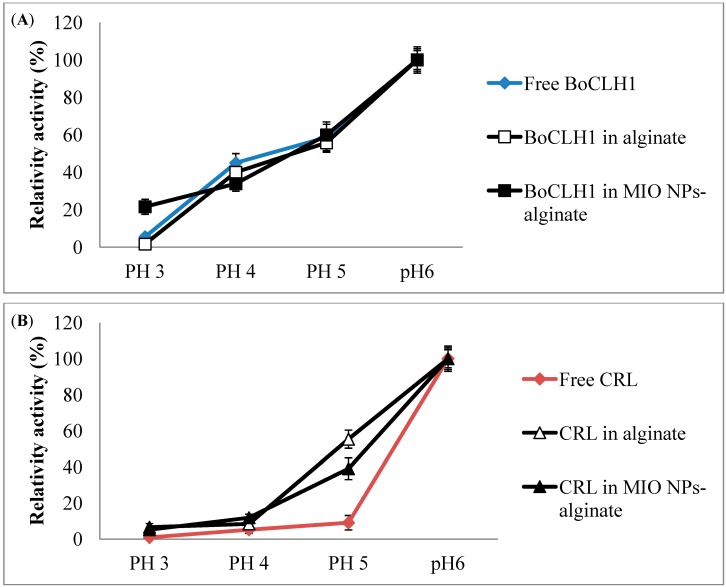
The pH effects on the activity of immobilized BoCLH1 and CRL activity. (**A**) The pH activity of free BoCLH1 (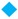), BoCLH1 in alginate (□) and BoCLH1 in MIO NPs-alginate (■) indicated, respectively. (**B**) The pH activity of free CRL (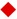), CEL in alginate (△) and CRL in MIO NPs-alginate (▲) indicated, respectively. The Chlase and esterase assay using Chl a and *p*-nitrophenol butyrate as substrate respectively. Relative activity (%) was triplicate measured according to the standard Chlase and esterase assay.

The Chlase activity of free and immobilized enzyme preparations was measured at 40 °C at different pH values ranging from 3 to 6. The results are shown in Figure 5A. The optimum pH of all preparations was pH 6. We defined pH 6 as 100%. The pH activity profiles of BoCLH1 entrapped in alginate and MIO NP-alginate composite beads were relatively similar to that of the soluble enzyme at pH 4 to 6. However, the BoCLH1 entrapped in MIO NP-alginate composite beads retained 22% relative activity at pH 3. It seems that the presence of MIO NPs enhances the protective effect on the BoCLH1 under pH 3 conditions. The loss of activity was most probably due to the conformational change in the enzyme structure. This result could be attributed to the fact the MIO NPs probably postpone the enzyme structural denaturation. Previous studies indicated that enzymes directly immobilized onto MIO NPs exhibited better pH tolerance [39,40]. Thus we believe that the alginate used entrapped the enzyme by covalent linkage onto MIO and this could increase the resistance against conformational changes.

In addition, the esterase assay of free CRL and immobilized enzyme preparations was measured at 37 °C for 10 min. Free CRL activity sharply decreased in an acidic environment. The results presented in Figure 5B show that the relative activity of free CRL was reduced to 9% at pH 5. The immobilized CRL demonstrated higher tolerance than free CRL at pH 5. However, the relative activity of CRL entrapped in alginate (55% retained) was higher than in MIO NP-alginate composite beads (39%) at pH 5. This result could be attributed to the fact that MIO NPs probably limit the substrate access to the enzyme. The relative activity of free CRL was reduced to 5.2% at pH 4, while the CRL entrapped in alginate and MIO NP-alginate composite beads showed 8.4% and 11.2% relative activity at pH 4, respectively. Under pH 3 conditions, free CRL retained 0.9% relative activity, however, the CRL entrapped in alginate and MIO NP-alginate composite beads retained 6.6% and 5.2% relative activity. As shown in Figure 5, the alginate beads and alginate composite beads showed excellent acid resistance characteristics. The greater stability of the immobilized enzyme may be ascribed to the stabilizing effects of alginate protection.

### 2.6. Repeated Use of Immobilized Enzymes

**Figure 6:**
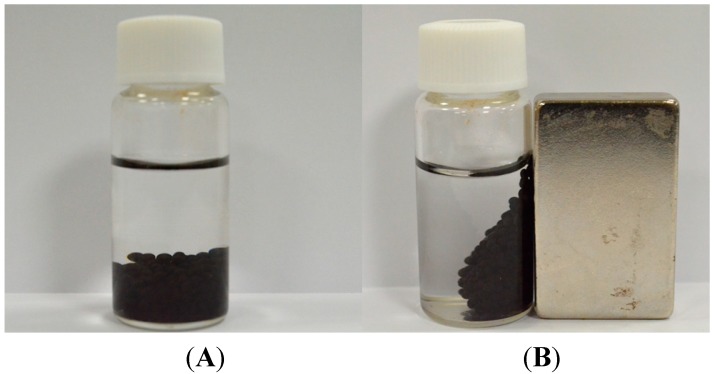
The synthesized MIO entrapped alginate beads were attracted by a magnet, indicating their superparamagnetic characteristics. (**A**) The MIO entrapped alginate beads distributed in suspension (**B**) The MIO entrapped alginate beads could be very easily and efficiently separated from the suspension using a magnet.

**Figure 7:**
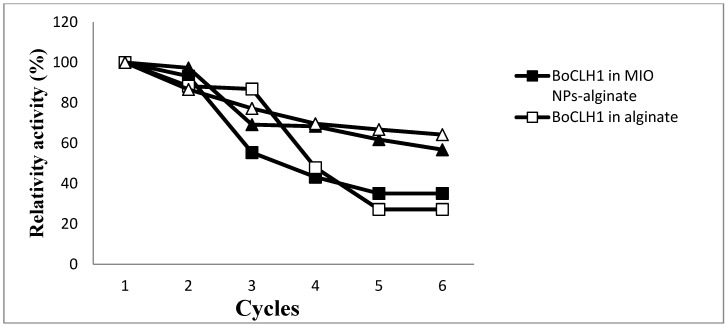
The residual activity of the BoCLH1 in the alginate (□) and MIO NP-alginate (■) and CRL entrapped in the alginate (△) and MIO NP-alginate (▲) at pH 6 in reaction buffer for 30 min each cycle.

To test the stability of BoCLH1 and CRL entrapped in the alginate or MIO NP-alginate beads, the beads were used six times for catalytic reaction. The enzyme immobilized MIO NPs-alginate beads could be collected by an external magnet (Figure 6). Each reaction lasted 30 min. After the test, the alginate and MIO NP-alginate beads were recovered by filtration and a magnet, respectively. The reaction medium was replaced with fresh medium. We defined first use as 100%. Figure 7 indicates the results of repeated use of the alginate and MIO NP-alginate bead entrapped enzyme. Comparing MIO NP-alginate BoCLH1and alginate BoCLH1, the third use brought about a 45% activity loss in MIO NP-alginate BoCLH1, however, alginate BoCLH1 retains 86% of the activity. Moreover, MIO NP-alginate BoCLH1 retains 43% or 35% activity after the fourth or fifth cycle, the activity of alginate BoCLH1 decreases to 48% at the fourth use and the activity is further reduced (27%) after the fifth and sixth use. In addition, CRL entrapped in the alginate and MIO NP-alginate beads showed higher stability (56%–80%) for catalytic reactions after the third cycle.

## 3. Experimental Section

### 3.1. Materials

Sodium alginate (cat. A2158), iron(II) chloride tetrahydrate (FeCl_2_•4H_2_O, 99%), iron (III) chloride hexahydrate (FeCl_3_•6H_2_O, 98%), and sodium hydroxide (NaOH) were purchased from Sigma-Aldrich (Sigma, St. Louis, MO, USA), J. T. Baker (Center Valley, PA, USA), Alfa Aesar (Ward Hill, MA, USA), Mallinckrodt (Hazelwood, MO, USA), and Nihon Shiyaku Reagent (Tokyo, Japan), respectively, and were all used as received without further processing.

### 3.2. Enzyme Preparation

Recombinant *E. coli* (BL21) harboring plasmid pET51b-BoCLH1 containing the gene encoding *BoCLH1* was used for the expression of the recombinant BoCLH1. *E. coli* cells were grown in 50 mL Luria-Bertani (LB) medium containing 100 g/mL ampicillin at 37 °C for 16 h. Protein overexpression was induced by adding isopropyl β-D-1-thiogalactopyranoside (IPTG) to a final concentration of 0.4 mM into the refreshed 1 L LB medium culture at 16 °C overnight. Upon centrifugation (13,000 rpm for 10 min at 4 °C), the harvested cell pellet (from 1 L LB broth culture) was suspended in 10 mL TE buffer (100 mM Tris·HCl, 1 mM EDTA, pH 8) and then was frozen at −70 °C for 30 min. After thawing, the suspension was sonicated and then centrifuged (13,000 rpm for 20 min at 4 °C). The sonication conditions were 10 pulses at 30 s each with 30 s intervals set at 2.5 (∼137.5 W) (Sonicator XL-2020, Misonix Co., New York, NY, USA). The crude cell lysate containing the enzyme was collected by centrifugation at 13,000 rpm for 20 min and filtered with 0.22 μm filter. *Candida rugosa* lipase (CRL, Sigma L-1754) was dissolved in TE buffer.

### 3.3. Synthesis of Magnetic Iron Oxide Nanoparticles

**Figure 8:**
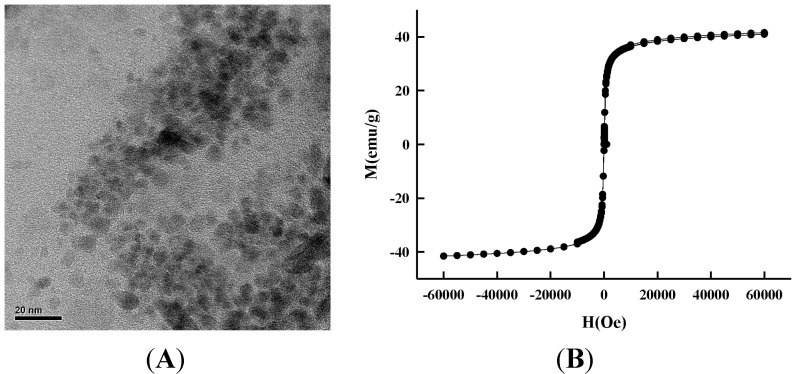
(**A**) The TEM photo of the synthesized MIO NPs. (**B**) The SQUID data of the synthesized MIO nanoparticles.

FeCl_2_•4H_2_O (4.776 g, dissolved in 12 mL of 2N HCl solution) and FeCl_3_•6H_2_O (3.24 g, dissolved in 12 mL of 2 N HCl solution) respectively. Glycine (0.5 g, dissolved in 5 mL dd-water) was used as a protector. FeCl_2_ solution (1 mL), FeCl_3_ (4 mL) and glycine (5 mL) were mingled to obtain an iron-containing solution. A NaOH solution (20%, 3 mL) was gradually added into the iron solution with constant stirring (900 rpm) for 20 min. The obtained precipitate was then washed with dd-H_2_O to get the MIO NPs [41,42,43,44]. A transmission electron microscopy (TEM) photo and SQUID data of the synthesized MIO NPs are shown in Figure 8.

### 3.4. Synthesis of MIO NPs-Alginate Beads

**Figure 9:**
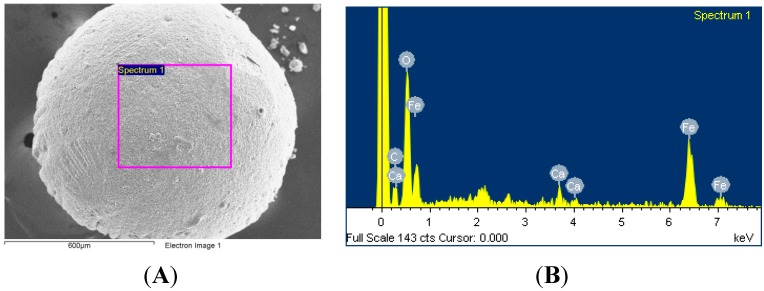
(**A**) The SEM photo of the synthesized MIO NPs-alginate bead. (**B**) The EDS analysis of the synthesized MIO NPs-alginate bead.

Alginate solution (5%, 3 mL), MIO solution (2 mL) and dd-H_2_O (3 mL) were mixed with constant stirring for 5 min. The solution was then dropped into a CaCl_2_ solution (20 wt%) by means of a syringe and pump, and MIO NP-encapsulated alginate beads were observed. These spheres were collected by centrifugation and washed twice with dd-H_2_O (30 mL) to remove the residues. A scanning electron microscope (SEM) photo and the energy-dispersive X-ray spectroscopy (EDS) analysis of the synthesized MIO NPs-alginate beads are shown in Figure 9.

### 3.5. Preparation of Enzyme Encapsulated in Magnetic Alginate Composite Beads

Enzyme (chlorophyllase or esterase) (3 mL), alginate solution (5%, 3 mL), MIO NPs solution (2 mL) and dd-H_2_O (3 mL) were mixed through constant stirring for 5 min. The solution was then dropped into a CaCl_2_ solution (20 wt%) by means of a syringe and pump. Enzyme (BoCLH1 or CRL)-embedded magnetic alginate composite beads were then observed. The fabricated spheres were collected by centrifugation and washed twice with 30 mL dd-water to remove the non-entrapped enzymes and residues.

### 3.6. Chlorophyllase Assay of the Immobilized BoCLH1

Immobilized BoCLH1 (0.1 g), 130 μL of reaction buffer (100 mM sodium phosphate, pH 6, and 0.24% Triton X-100), and 15 μL Chl a (from *Anacystis nidulans* algae; Sigma, St. Louis, MO, USA) were dissolved in ethanol (at a final concentration of 500 μM). The reaction mixture was incubated in a shaking water bath at 40 °C. The amount of product formed had a linear relationship with reaction time within 30 min. Therefore, in the following assay we carried out the reaction for 30 min to measure the initial velocity. Enzyme reaction was stopped by transferring the reaction mixture to a centrifuge tube containing 1 mL of stop reaction buffer (ethanol/hexane/10 mM KOH = 4:6:1 (v/v)). The mixture was vigorously vortexes and centrifuged at 12,000 rpm for 2 min for phase separation. Collecting the aqueous ethanol layer (Chlide a remained). The absorbance of the aqueous ethanol phase was measured at 667 nm for Chlide a with a spectrophotometer. The amount of each product in the ethanol layer was estimated from the millimolar extinction coefficient of 81 mM^−1^ cm^−1^ for Chlide a [34].

### 3.7. Esterase Assay of the Immobilized CRL

The esterase activity was assayed by a Synergy^TM^ HT spectrophotometer (BioTek, Winooski, VT, USA). The hydrolysis of *p*-NP butyrate was carried out at 37 °C in 500 μL of 50 mM sodium phosphate buffer (pH 6) containing 0.5% Triton X-100 and a 5 mM solution of the corresponding *p*-nitrophenol butyrate (Sigma). The reaction mixture was transferred to a 10 mm cuvette. The increase in absorbance was recorded for 10 min at 348 nm (isosbestic point of the *p*-nitrophenol/*p*-nitrophenoxide couple). One unit of activity was defined as the quantity of enzyme necessary to release 1 μmol of *p*-nitrophenol per minute under the above conditions [35].

### 3.8. Operational Stability

The operational stability of the immobilized enzyme was monitored by determining the residual activity of the immobilized enzyme after each cycle. The reaction was allowed to proceed as described for the chlorophyllase or esterase assays. After each cycle, the immobilized enzyme was recovered by filtration or a magnet.

### 3.9. The pH Effects of Immobilized BoCLH1 and CRL Activity

The pH effects on immobilized BoCLH1 and CRL were assessed using Chl a and *p*-NP butyrate as a substrate. The optimal pH of immobilized enzymes was investigated in the pH range of 3–5 using 50 mM sodium acetate buffer and pH range of 6 using Good’s buffer (50 mM each of Bicine, CAPS and bis-Tris propane). BoCLH1 residual activity was checked at 40 °C for 30 min and CRL residual activity was determined spectrophotometrically at 37 °C for 10 min under the standard test conditions.

### 3.10. Instruments

Size distributions of the various droplets samples were obtained from the random sampling of about 50 individual spheres to minimize selection bias. An inverted microscope system, including an optical microscope (BX60, Olympus, Tokyo,, Japan) and a digital camera (DP70, Olympus, Tokyo,, Japan), were employed for imaging. The average diameter of the beads, expressed as mean ± standard deviation, was obtained from the photomicrograph. A BioTek Synergy^TM^ HT spectrophotometer was used to assay the enzyme activity.

## 4. Conclusions

We have proposed a facile approach for the immobilization of enzymes in MIO NP-alginate beads that can encapsulate *Brassica oleracea* chlorophyllase 1 (BoCLH1) and *Candida rugosa* lipases (CRL). Currently the diameters of the fabricated beads are about 1.6 mm to 2.5 mm, but the particle size could be reduced significantly in the future by employing electrostatic or microfluidic droplet technology. The excellent acid-resistant characteristics of alginate ensured that the immobilized enzymes maintain the enzymatic properties in the acidic environment. The MIO NP-alginate composite beads could be collected using an external magnet after catalytic reaction and they were reusable for at least six cycles. The results suggest that the prepared enzyme immobilized MIO NP-alginate beads have potential for industrial applications in the future.
