# Correction: Yang, C.-H., *et al.* Immobilization of *Brassica oleracea* Chlorophyllase 1 (BoCLH1) and *Candida rugosa* Lipase (CRL) in Magnetic Alginate Beads: An Enzymatic Evaluation in the Corresponding Proteins. *Molecules* 2014, *19*, 11800-11815

**DOI:** 10.3390/molecules20047325

**Published:** 2015-04-21

**Authors:** Chih-Hui Yang, Chih-Chung Yen, Jyun-Jen Jheng, Chih-Yu Wang, Sheau-Shyang Chen, Pei-Yu Huang, Keng-Shiang Huang, Jei-Fu Shaw

**Affiliations:** 1 Department of Biological Science & Technology, I-Shou University, Kaohsiung 840, Taiwan; 2 Department of Biomedical Engineering, I-Shou University, Kaohsiung 840, Taiwan; 3 The School of Chinese Medicine for Post-Baccalaureate, I-Shou University, No. 8, Yida Road, Jiaosu Village Yanchao District, Kaohsiung 82445, Taiwan

The authors wish to correct Scheme 1, and Figure 1, Figure 4 and Figure 7 in [1] as follows.

Scheme 1 should include phytol and fatty acid.

**Scheme 1:**
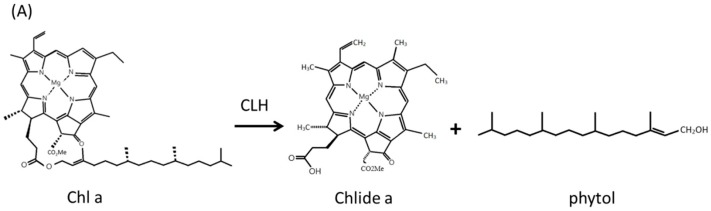

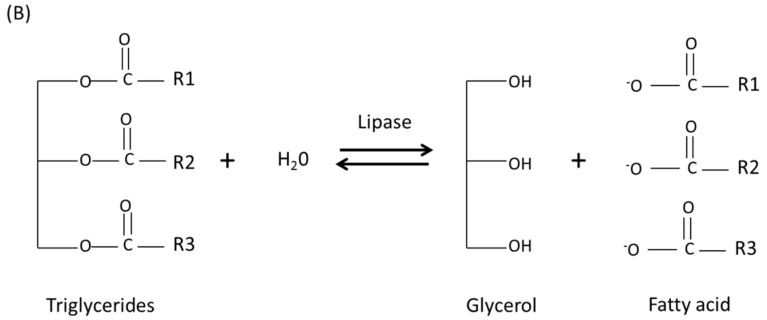
Enzymatic reaction of Chlorophyllase (CLH) and lipase. (**A**) Chlorophyllase catalyzes the hydrolysis of chlorophyll a (Chl a), chlorophyllide (Chlide a) and phytol. (**B**) Lipase catalyzes hydrolysis or synthesis of a triglycerol.

In Figure 1 Alginate should replace Chitosan:

**Figure 1:**
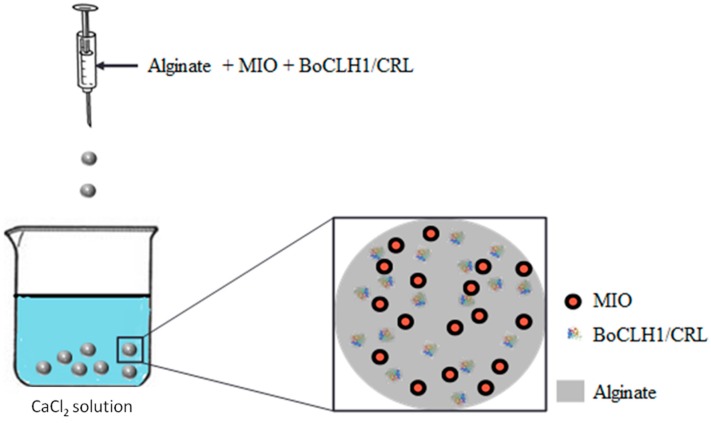
Schematic diagram showing the synthesis of enzyme encapsulated magnetic alginate composite beads. MIO is magnetic iron oxide (Fe_3_O_4_ nanoparticles); BoCLH1 is *Brassica oleracea* chlorophyllase 1; CRL is *Candida rugosa* lipase.

Figure 4A,B should be replaced. The correct Figure 4 is as follows: 

**Figure 4:**
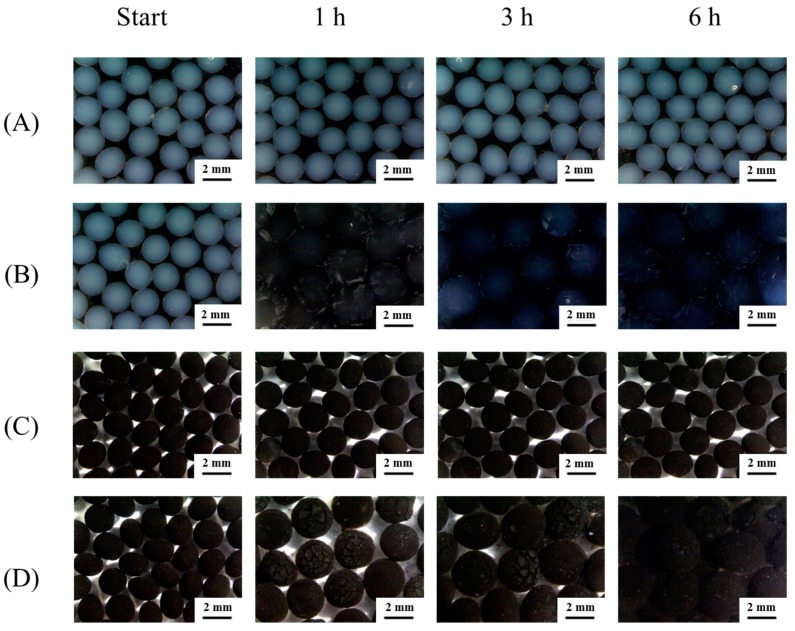
The degradation of alginate beads in various pH environments.

In Figure 7 CRL entrapped in the alginate (∆) and MIO NP-alginate (▲) should be labeled: 

**Figure 7:**
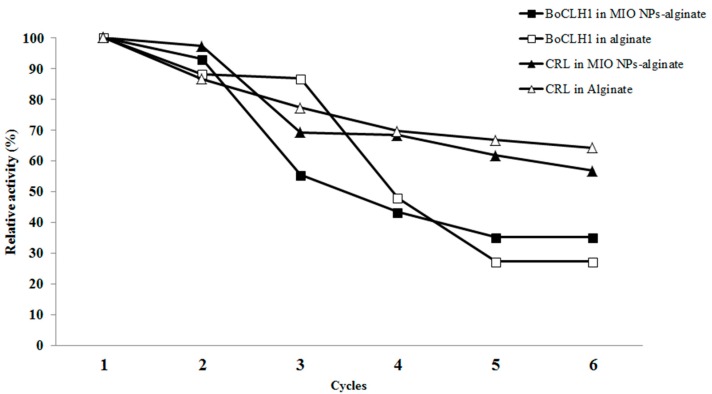
The residual activity of the BoCLH1 in the alginate (□) and MIO NP-alginate (■) and CRL entrapped in the alginate (∆) and MIO NP-alginate (▲) at pH 6 in reaction buffer for 30 min each cycle.

The authors would like to apologize for any inconvenience caused to the readers by these changes. The article will be updated on the journal website, with the original version remaining available at the same location.
